# Menopausal Symptom Burden, Quality of Life, Healthcare-Seeking Behavior, and Treatment Satisfaction: A Cross-Sectional Study of Romanian Women

**DOI:** 10.3390/healthcare14172693

**Published:** 2026-08-24

**Authors:** Andrei Pescaru, Flavia Crișan, Radu Ioan Lala, Adrian Cosmin Ilie, Norbert Wellmann, Adelina Marițescu, Alaviana Monique Faur, Daian Ionel Popa, Roxana Folescu

**Affiliations:** 1 Doctoral School, “Victor Babes” University of Medicine and Pharmacy, 300041 Timisoara, Romania; andrei.pescaru@umft.ro (A.P.); norbert.wellmann@umft.ro (N.W.); adelina.maritescu@umft.ro (A.M.); alaviana.faur@umft.ro (A.M.F.); 2 University Clinic Toxicology, Pharmaceutical Industry, Management and Legislation, Research Centre for Pharmaco-Toxicological Evaluations, Faculty of Pharmacy, “Victor Babes” University of Medicine and Pharmacy, 300041 Timisoara, Romania; 3 Department of Cardiology, Arad Western University Vasile Goldis, 310414 Arad, Romania; lala.radu@uvvg.ro; 4 Department of Functional Sciences III, Division of Public Health and History of Medicine, Centre for Translational Research and Systems Medicine, Faculty of Medicine, “Victor Babes” University of Medicine and Pharmacy, 300041 Timisoara, Romania; ilie.adrian@umft.ro; 5 Center for Research and Innovation in Personalized Medicine of Respiratory Diseases, “Victor Babes” University of Medicine and Pharmacy, 300041 Timisoara, Romania; 6 Pulmonary Rehabilitation Center, Clinical Hospital of Infectious Diseases and Pulmonology “Victor Babes”, 300310 Timisoara, Romania; 7 Research Center for Medical Communication, “Victor Babes” University of Medicine and Pharmacy, 300041 Timisoara, Romania; daian-ionel.popa@umft.ro; 8 Department of Balneology, Medical Recovery and Rheumatology, Family Medicine University Clinic, Center for Preventive Medicine, Center for Advanced Research in Cardiovascular Pathology and Hemostaseology, “Victor Babes” University of Medicine and Pharmacy, 300041 Timisoara, Romania; folescu.roxana@umft.ro

**Keywords:** menopause, patient-reported outcome measures, quality of life

## Abstract

**Highlights:**

**What are the main findings?**

- Higher BMI, postmenopausal stage, greater comorbidity burden, and use of medication for menopausal symptoms were independently associated with poorer menopause-specific quality of life.
- Women who sought medical care reported greater symptom burden and poorer quality of life than non-seekers. Consultation delay was not significantly associated with symptom burden or quality of life among women who had sought care.

**What are the implications of the main findings?**

- Menopause assessment should combine symptom and quality-of-life measures with clinical context, including BMI, menopausal stage, comorbidity burden, psychological concerns, and current treatment.
- Persistent symptoms or poor quality of life among women already using menopause medication may indicate a need to review treatment type, adherence, expectations, and unmet needs; however, the cross-sectional findings do not establish treatment effectiveness or causality.

**Abstract:**

**Background/Objectives**: Menopausal symptoms can impair quality of life and influence healthcare use. This study examined symptom burden, menopause-specific quality of life, healthcare seeking, consultation delay, treatment satisfaction, and associated demographic and clinical factors among Romanian women. **Methods**: This cross-sectional study included 204 peri- and postmenopausal women. We assessed symptoms and quality of life using the Menopause Rating Scale (MRS) and the Menopause-Specific Quality of Life Questionnaire (MENQOL). We compared groups, ran Spearman correlations, and used multivariable linear regression to identify demographic and clinical factors associated with MENQOL. We excluded MRS from the regression because of construct overlap, and restricted delay analyses to care seekers. **Results**: Care seekers had higher MRS (18.0 vs. 14.5; *p* = 0.041) and MENQOL scores (3.95 vs. 3.26; *p* = 0.009). MRS and MENQOL were strongly correlated (ρ = 0.858; *p* < 0.001), but consultation delay was not associated with either outcome among care seekers. In the adjusted model, higher BMI, postmenopausal stage, greater comorbidity burden, and menopause-medication use were associated with poorer quality of life (adjusted R^2^ = 0.138). Treatment-satisfaction correlations were inverse but exploratory. **Conclusions**: Menopause-specific quality of life varied according to body composition, reproductive stage, general health burden, and treatment context. Clinical assessment should integrate these factors and review persistent symptoms among treated women. The cross-sectional findings do not establish causality or treatment effectiveness.

## 1. Introduction

Menopause is a universal biological change marked by the permanent end of ovarian activity and menstruation. While it is a normal part of aging, it often brings vasomotor, psychological, physical, and genitourinary symptoms that can significantly affect quality of life, daily activities, and overall well-being [1,2,3,4].

Despite widespread awareness of menopausal symptoms and their significant impact, many women avoid seeking medical help or receive inadequate treatment due to limited knowledge, concerns about treatment options, or the belief that symptoms should be endured. This can lead to persistent symptoms and a decline in quality of life [5,6].

Recent clinical guidelines and menopause research have increasingly prioritized patient-centered care by acknowledging women’s symptom experiences, quality of life, treatment preferences, and shared decision-making as key aspects of menopause management [1,7,8].

Previous studies have examined menopausal symptom severity, menopause-specific quality of life, healthcare-seeking behavior, consultation delay, and treatment satisfaction, either separately or in selected combinations [3,5,9,10].

However, fewer studies have assessed all these patient-reported dimensions concurrently within the same cohort, particularly in the Romanian healthcare context. The present study therefore contributes primarily by integrating and describing these dimensions, rather than identifying a novel biological or causal relationship [7,8,9,10].

This study aimed to examine the associations among menopausal symptom burden, menopause-specific quality of life, healthcare-seeking behavior, consultation delay, and treatment satisfaction, and to identify demographic and clinical factors independently associated with menopause-specific quality of life within a cohort of Romanian peri- and postmenopausal women.

## 2. Materials and Methods

### 2.1. Study Design and Setting

This cross-sectional observational study assessed menopausal symptom burden, menopause-specific quality of life, healthcare-seeking behavior, consultation delay, and treatment satisfaction among peri- and postmenopausal women. We recruited participants between February and June 2026 through two pathways. Hospital-based participants were current or former patients of the Department of Obstetrics and Gynecology at the Municipal Emergency Clinical Hospital of Timișoara, Romania, whereas community participants were recruited through community contacts and personal networks. Of the 204 participants, 78 (38.2%) were recruited through the hospital or clinical setting and 126 (61.8%) through community-based recruitment. The two pathways were intended to include women with different levels of engagement with healthcare services.

Eligible women completed a structured questionnaire that captured sociodemographic data, clinical features, menopausal symptoms, healthcare utilization, and treatment outcomes. The study was designed and reported in accordance with the STROBE guidelines for cross-sectional research [11].

The study protocol received approval from the management of the Municipal Emergency Clinical Hospital of Timișoara (Approval No. E-891/27.02.2026). All participants provided written informed consent before enrollment. The study adhered to the Declaration of Helsinki and relevant European data protection laws [12].

### 2.2. Participants

Women were eligible if they were aged 40 years or older, were in early or late perimenopause or postmenopause, could understand and complete the questionnaires in Romanian, and provided written informed consent. We determined menopausal stage from the original categorical questionnaire response based on menstrual history. Women were ineligible if they were younger than 40 years, declined participation, or had cognitive or communication difficulties that prevented questionnaire completion. All 204 submitted questionnaires contained sufficient information to calculate the MRS and MENQOL scores and were included in the analysis. We determined the sample size to detect a weak correlation between variables in the menopausal care pathway and patient-reported outcomes. With a two-sided α of 0.05, 80% power, and an anticipated correlation coefficient of 0.20, we needed at least 194 participants. The study ultimately included 204 women, surpassing the minimum required sample size.

### 2.3. Data Collection and Study Variables

Sociodemographic and clinical variables included age, BMI, residence, education, marital status, menopausal stage, comorbidity count, use of medication for menopausal symptoms, use of an SSRI or anxiolytic, and recruitment source. Hospital- and community-based recruitment was intended to include women with different levels of healthcare engagement; however, because neither pathway used population-based probability sampling, we could not eliminate selection bias. We used standardized, validated questionnaires to assess menopausal symptom burden and menopause-specific quality of life. We evaluated healthcare-seeking behavior, consultation delay, and treatment satisfaction using study-specific questionnaire items. Because all data were self-reported, recall and reporting biases could not be entirely avoided.

### 2.4. Outcome Measures

#### 2.4.1. Menopause Rating Scale (MRS)

Menopausal symptom severity was assessed using the Menopause Rating Scale (MRS), a validated tool designed for patients to report the severity of menopause-related symptoms. It includes 11 items divided into three domains: somatic, psychological, and urogenital. Each item is rated on a five-point Likert scale from 0 (no symptoms) to 4 (very severe), with total scores ranging from 0 to 44. Higher scores indicate more severe symptoms. The MRS is well-validated, reliable, and responsive, and it is commonly utilized in both clinical settings and menopause research [13].

#### 2.4.2. Menopause-Specific Quality of Life Questionnaire (MENQOL)

Menopause-specific quality of life was evaluated using the MENQOL questionnaire, a validated tool comprising 29 items across four domains: vasomotor, psychosocial, physical, and sexual functioning. Participants initially indicated whether each symptom had been present or absent during the previous month. Absent symptoms were assigned a score of 1. When a symptom was present, participants rated how bothered they were on a scale from 0 (not at all bothered) to 6 (extremely bothered); these responses were converted to scores from 2 to 8. Domain scores were calculated as the mean of the converted item scores within each domain, and the MENQOL total score was calculated as the mean of all 29 converted item scores. Domain and total scores therefore ranged from 1 to 8, with higher scores indicating poorer menopause-specific quality of life [14].

#### 2.4.3. Healthcare-Seeking Behavior and Treatment Satisfaction

We evaluated healthcare-seeking behavior by asking whether participants had consulted a physician about menopausal problems. Participants also reported the time from symptom onset to consultation in months. The questionnaire instructed women who had not consulted a physician to record a delay of 0 months. Consequently, we restricted consultation-delay analyses to women who reported seeking medical care and provided an interpretable delay value. The questionnaire also instructed women who had not sought care to select the general practitioner option for the first-contact item; therefore, we did not interpret first-contact responses among non-seekers. We analyzed consultation delay continuously, without applying a categorical threshold.

Treatment satisfaction was assessed in all participants using the study-specific item: “Overall, how satisfied are you with the treatment or management received for your menopausal symptoms?” For women who had not sought formal medical care, management included any self-management strategies used for their menopausal symptoms. The original item was administered in Romanian. Responses were recorded on a five-point Likert scale: 1, very dissatisfied; 2, dissatisfied; 3, neither satisfied nor dissatisfied; 4, satisfied; and 5, very satisfied. Higher scores indicated greater treatment satisfaction. We developed this item for this study; it was not part of a validated treatment-satisfaction instrument and had not undergone separate psychometric evaluation. We did not calculate a composite score, and we analyzed responses as an ordinal variable.

### 2.5. Statistical Analysis

Statistical analyses were conducted with MedCalc Statistical Software, version 23.4.0 (MedCalc Software Ltd., Ostend, Belgium). We assessed the normality of continuous variables using the Shapiro–Wilk test and visual assessment of data distributions. Since most continuous variables were not normally distributed, results are presented as medians with interquartile ranges (IQRs), and categorical data are reported as frequencies and percentages.

Comparisons between women who sought medical help for menopausal symptoms and those who did not were performed using the Mann–Whitney U test. Menopause-medication users and non-users were compared using the same test. Relationships between MRS and MENQOL scores were examined using Spearman’s rank correlation coefficient (ρ), with corresponding 95% confidence intervals (CIs). Because participants who had not consulted a physician were instructed to record a consultation delay of 0 months, we restricted correlations involving consultation delay to women who reported seeking medical care and provided an interpretable delay value. We considered correlations involving treatment satisfaction exploratory because we assessed satisfaction using a nonvalidated, study-specific single item. To determine whether recruitment source modified the association between symptom burden and healthcare-seeking behavior, we ran a logistic regression among all 204 participants, including MRS total score, recruitment source, and their interaction.

To identify demographic and clinical factors independently associated with menopause-specific quality of life, we performed a multiple linear regression analysis with the MENQOL total score as the dependent variable. We entered the following variables simultaneously: age, BMI, menopausal stage, comorbidity count, university education, urban residence, married or partnered status, recruitment source, menopause-medication use, and SSRI or anxiolytic use. Early perimenopause, community recruitment, no university education, non-urban residence, not married or partnered, no menopause-medication use, and no SSRI or anxiolytic use were used as the respective reference categories. We did not include the MRS total score because MRS and MENQOL assess substantially overlapping symptom-related domains, which would make a model containing both measures conceptually tautological.

We reported regression coefficients (B), heteroscedasticity-consistent HC3 95% confidence intervals (CIs), *p*-values, and variance inflation factors (VIFs). Residual normality, homoscedasticity, functional form, and potentially influential observations were assessed using the Shapiro–Wilk test, Breusch–Pagan test, Ramsey RESET test, externally studentized residuals, leverage values, and Cook’s distance. We examined a log transformation of the MENQOL total score as a sensitivity analysis. All tests were two-sided, and we considered *p*-values less than 0.05 statistically significant.

No submitted questionnaire was excluded because all 204 participants had complete data for the MRS and MENQOL outcomes and for the variables included in the multivariable regression and recruitment-source analyses. We restricted consultation-delay analyses to the care seekers.

## 3. Results

We included all 204 submitted questionnaires in the final analysis. All participants had complete data for the MRS and MENQOL outcomes and for the variables included in the multivariable regression analysis. Table 1 summarizes baseline demographic, clinical, treatment, and healthcare-seeking characteristics.

Participants had a median age of 51 years (IQR 48–55), and 47.5% were postmenopausal. Most participants were recruited from the community (61.8%), while 38.2% were recruited through the hospital or clinical setting. Most lived in urban areas (71.6%), had a university education (64.7%), and were married or partnered (77.9%). Overall, 58.8% reported seeking medical help for menopausal symptoms. Among the care seekers, the median time from symptom onset to consultation was 6 months (IQR 2–12). Menopause medication was used by 21.1% of participants, and 10.3% reported using an SSRI or anxiolytic.

We evaluated menopausal symptom severity and menopause-specific quality of life with the MRS and MENQOL questionnaires. Table 2 displays the descriptive results for total scores and each questionnaire domain.

Participants had a median MRS total score of 17.0 (IQR 10.0–24.0), indicating substantial symptom burden. Somatic symptoms had the highest median score among the MRS domains, while psychological and urogenital symptoms had lower median scores. The median MENQOL total score was 3.66 (IQR 2.59–4.66). The most significant impact on menopause-related quality of life was in the sexual domain, followed by the physical domain. Vasomotor and psychosocial domains were less affected.

Participants were grouped based on whether they sought medical care for menopausal symptoms to assess differences in symptom burden and menopause-specific quality of life. Table 3 shows the comparison results between these groups.

Women who sought medical attention experienced a notably higher overall burden of menopausal symptoms compared to those who did not (median MRS total score 18.0 vs. 14.5, *p* = 0.041). This difference mainly stemmed from psychological symptoms, while somatic and urogenital symptom scores were similar across both groups. Additionally, women seeking medical care reported significantly worse menopause-related quality of life, indicated by higher scores in the MENQOL total, vasomotor, psychosocial, and physical domains. There was no significant difference observed in the sexual domain.

We performed correlation analyses to explore relationships among menopausal symptom burden, menopause-specific quality of life, healthcare delay, and treatment satisfaction. Table 4 summarizes the correlation coefficients.

The MRS total score was strongly correlated with the MENQOL total score (ρ = 0.858; *p* < 0.001), and each MRS domain was also strongly associated with MENQOL total. Given the overlapping symptom-related content of the two instruments, these coefficients indicate close correspondence between related patient-reported constructs and should not be interpreted as evidence that one measure independently predicts the other. Among the care seekers, consultation delay was not significantly correlated with either MRS total (ρ = 0.136; *p* = 0.141) or MENQOL total (ρ = 0.078; *p* = 0.401). Treatment satisfaction showed inverse correlations with symptom burden and quality-of-life impairment; however, these correlations are considered exploratory because satisfaction was measured using a nonvalidated single item.

We conducted a multivariable linear regression analysis to identify demographic and clinical factors independently associated with menopause-specific quality of life. We used the MENQOL total score as the dependent variable and considered age, BMI, menopausal stage, comorbidity count, university education, urban residence, married or partnered status, recruitment source, menopause-medication use, and SSRI or anxiolytic use simultaneously as independent variables. We did not include MRS because of its substantial conceptual overlap with MENQOL. Table 5 presents the findings. In unadjusted comparisons, women using medication for menopausal symptoms had higher MRS scores than non-users (21.0 [IQR 14.0–25.5] vs. 17.0 [IQR 9.0–22.0]; *p* = 0.017) and higher MENQOL scores (4.41 [IQR 3.24–4.91] vs. 3.55 [IQR 2.48–4.45]; *p* = 0.009).

In the adjusted model, higher BMI was associated with a higher MENQOL total score, indicating poorer menopause-specific quality of life (B = 0.064; HC3 95% CI 0.006–0.122; *p* = 0.032). Compared with early perimenopausal women, postmenopausal women had higher adjusted MENQOL scores (B = 0.747; 95% CI 0.200–1.294; *p* = 0.008), whereas late perimenopause was not significantly associated with MENQOL. Greater comorbidity count (B = 0.209; 95% CI 0.026–0.392; *p* = 0.025) and menopause-medication use (B = 0.563; 95% CI 0.125–1.001; *p* = 0.012) were also independently associated with higher MENQOL scores. Age, education, residence, marital status, recruitment source, and SSRI or anxiolytic use were not significantly associated with MENQOL. The model explained 18.4% of the variance in MENQOL total score. All VIF values were low (range 1.02–2.12), indicating no evidence of problematic multicollinearity.

Diagnostic assessment supported the revised model. Residuals did not depart significantly from normality (Shapiro–Wilk W = 0.997; *p* = 0.958), and neither the Breusch–Pagan test (*p* = 0.790) nor the Ramsey RESET test (*p* = 0.962) indicated heteroscedasticity or functional-form misspecification. Eleven observations exceeded the conservative Cook’s distance screening threshold of 4/n, although the maximum Cook’s distance was 0.111. Eight observations exceeded the leverage screening threshold of 2 k/n, with a maximum leverage value of 0.181. No externally studentized residual exceeded an absolute value of 3. Considered together, these findings did not indicate substantial individual influence or major model misspecification. Log transformation of the MENQOL total score worsened residual normality (Shapiro–Wilk *p* < 0.001) and was therefore not retained.

Recruitment source did not significantly modify the association between MRS total score and healthcare-seeking behavior. The interaction between MRS and hospital/clinical recruitment was not statistically significant (interaction OR = 0.989; 95% CI 0.922–1.060; *p* = 0.752). Among community-recruited women, the median MRS score was 15.5 (IQR 8.25–24.0) in non-seekers and 21.0 (IQR 13.75–26.25) in care seekers (*p* = 0.074). Among hospital- or clinically recruited women, the corresponding median scores were 14.0 (IQR 6.25–19.0) and 16.0 (IQR 10.0–21.0), respectively (*p* = 0.388).

## 4. Discussion

This cross-sectional study identified several demographic and clinical factors associated with menopause-specific quality of life among Romanian peri- and postmenopausal women. Higher BMI, postmenopausal stage, greater comorbidity burden, and use of medication for menopausal symptoms were independently associated with higher MENQOL scores. Women who sought medical care reported greater symptom burden and poorer menopause-specific quality of life than non-seekers. By contrast, consultation delay was not significantly associated with MRS or MENQOL among women who had sought care. Treatment satisfaction showed inverse exploratory correlations with symptom burden and quality-of-life impairment. We interpreted the strong MRS–MENQOL correlation descriptively because the two instruments overlap in symptom content.

MRS and MENQOL total scores were strongly correlated, consistent with previous observational evidence linking menopausal symptoms to poorer menopause-specific quality of life [2,3,15,16,17,18].

However, both instruments assess overlapping somatic, psychological, and urogenital or sexual symptom-related domains [13,14]. Their shared content and self-reported format likely contributed substantially to the magnitude of the correlation. The finding should therefore be interpreted as correspondence between related patient-reported constructs rather than evidence that MRS independently predicts a distinct MENQOL outcome. For this reason, we excluded MRS from the revised multivariable MENQOL model.

The MENQOL sexual domain did not differ significantly between women who sought medical care and those who did not. Because sexual well-being in midlife reflects biological, psychological, relational, and sociocultural factors beyond menopausal symptoms alone, this domain may be less closely related to menopause-specific healthcare-seeking behavior [2,14,19].

Women who sought medical care reported greater menopausal symptom burden and poorer menopause-specific quality of life than women who did not seek care. This pattern is consistent with international studies reporting that greater symptom severity is associated with healthcare seeking [5,6,9,20]. Recruitment source did not significantly modify the relationship between MRS and healthcare-seeking behavior, suggesting that the observed association did not differ detectably between community- and hospital-recruited participants. Nevertheless, the subgroup analyses had limited statistical power, and the cross-sectional design does not establish whether greater symptom burden preceded healthcare use or whether healthcare engagement influenced symptom reporting.

Greater symptom burden and poorer menopause-specific quality of life were associated with lower treatment satisfaction. However, satisfaction was measured using a single, study-specific ordinal item whose reliability, construct validity, and sensitivity to change are unknown. Although Spearman correlation suits ordinal rankings, it does not address measurement unreliability or establish that the item captures the multidimensional construct of treatment satisfaction. These correlations should therefore be considered exploratory and hypothesis-generating and should not be interpreted as evidence of treatment effectiveness. Lower reported satisfaction may reflect persistent symptoms, unmet expectations, limited treatment options, or communication difficulties, but these explanations were not directly evaluated [5,7,8,10,21,22].

Previous studies have examined these patient-reported dimensions separately or in selected combinations [5,6,9,10,15,16,17,18,20]. The contribution of the present study is therefore contextual and integrative, describing them concurrently within a Romanian cohort, rather than identifying a new clinical pathway or biological mechanism [1,7,8,23,24].

Higher BMI was independently associated with greater menopause-specific quality-of-life impairment. Because of the cross-sectional design, this finding does not establish that higher BMI causes poorer quality of life. The association may also reflect behavioral, metabolic, or other unmeasured factors and should therefore be interpreted cautiously. Postmenopausal women had higher adjusted MENQOL scores than women in early perimenopause, whereas late perimenopause did not differ significantly from early perimenopause. Women within the same menopausal stage may experience different symptom profiles, coping mechanisms, and degrees of quality-of-life impairment, highlighting the variability of the menopausal transition [2,3,4,8]. These observations support individualized assessment based on women’s symptoms, quality of life, and clinical needs rather than menopausal stage alone [1,7,8].

The between-group difference in symptom burden was most evident in the psychological MRS domain. Use of an SSRI or anxiolytic was reported by 21 women (10.3%); however, the study did not collect information on formal diagnoses of depression or anxiety. Medication use cannot establish a psychiatric diagnosis and may also reflect other clinical indications. Consequently, it is necessary to interpret the psychological-domain difference cautiously. Nevertheless, the finding supports consideration of psychological symptoms alongside vasomotor and physical symptoms when assessing women’s menopause-related concerns [2,3,17,18].

Age was not independently associated with the MENQOL total score in the revised multivariable model. Previous research suggests that women’s experiences of menopause may vary according to psychosocial factors, resilience, physical activity, education, and perceptions of aging [18,23,24,25]. However, the present cross-sectional findings do not support an independent age-related association after adjustment for the included demographic and clinical factors.

Greater comorbidity count was independently associated with poorer menopause-specific quality of life. This finding emphasizes that menopause-related quality of life should be interpreted within the context of women’s overall health burden rather than as an isolated consequence of reproductive stage. Previous studies have similarly shown that demographic and clinical characteristics may contribute to differences in menopause-related quality of life [15,18,26]. However, because the present study recorded only the number of comorbidities, it could not evaluate whether particular conditions had stronger associations with MENQOL.

Women using medication for menopausal symptoms had higher MRS and MENQOL scores than non-users, and menopause-medication use remained associated with poorer MENQOL in the adjusted model. The questionnaire asked about any medication used for menopausal symptoms and did not distinguish hormone therapy from other treatment types. These findings should not be interpreted as evidence that menopause medication worsens symptoms or quality of life. Confounding by indication is likely because women with more severe or persistent symptoms may be more likely to receive treatment. Evaluating treatment effectiveness would require longitudinal information on treatment type, indication, duration, adherence, and symptom severity before treatment initiation.

This study has several strengths. It assessed multiple aspects of menopause care from the patient’s perspective, including symptom severity, menopause-specific quality of life, healthcare-seeking behavior, consultation delay, and treatment satisfaction within the same cohort. It also used the validated and widely accepted MRS and MENQOL instruments [13,14,27]. Furthermore, the revised multivariable model evaluated demographic and clinical factors associated with MENQOL while avoiding the inclusion of MRS as an overlapping explanatory measure. The model incorporated BMI, menopausal stage, comorbidity burden, socioeconomic and demographic characteristics, recruitment source, and relevant medication use.

This study has several limitations. First, its cross-sectional design prevents determining temporal or causal relationships among menopausal symptoms, quality of life, healthcare-seeking behavior, treatment use, and satisfaction. All information was self-reported and may therefore have been affected by recall and reporting biases. We assessed treatment satisfaction using a single study-specific item that had not undergone psychometric validation. Its reliability and construct validity are unknown, and the observed satisfaction correlations should be interpreted as exploratory rather than as evidence of treatment effectiveness.

The MRS and MENQOL assess overlapping symptom-related domains and share the same self-reported measurement format. Consequently, their strong correlation may reflect shared content and common-method variance rather than an association between independent constructs. The medication question did not distinguish hormone therapy from other treatments for menopausal symptoms, and information regarding treatment duration, adherence, or pretreatment symptom severity was unavailable. Formal diagnoses of depression and anxiety were not collected, although SSRI or anxiolytic use was recorded. Finally, we recruited participants through two non-probability pathways within a single country, which may have introduced selection bias and limited generalizability to other populations and healthcare systems.

Although the revised regression model included BMI, education, marital status, residence, recruitment source, medication use, and other relevant demographic and clinical variables, residual confounding remains possible. Factors such as income, physical activity, diet, healthcare access, health literacy, detailed psychiatric history, treatment type and duration, and the specific nature or severity of comorbidities were not available. In addition, the model explained only a modest proportion of MENQOL variability and should be interpreted as an associative model rather than a comprehensive predictive model of menopause-specific quality of life. The findings support assessing menopause-specific quality of life within a broader clinical context that includes BMI, menopausal stage, comorbidity burden, current treatment, and psychological concerns. Persistent symptoms or poor quality of life among women already using medication should prompt a review of treatment type, adherence, expectations, and unmet needs rather than assumptions regarding treatment effectiveness. Combining symptom and quality-of-life assessment with these clinical factors may help identify women requiring more detailed evaluation, individualized counseling, or follow-up. These implications align with current recommendations for patient-centered, individualized menopause care [1,7,8,23,24,28,29,30].

Future longitudinal research should examine temporal relationships among symptom severity, menopause-specific quality of life, healthcare-seeking behavior, treatment exposure, and satisfaction. Such studies should use validated multidimensional satisfaction instruments; document treatment type, indication, duration, adherence, and baseline symptom severity; and collect formal psychiatric diagnoses alongside validated psychological assessments. Multicenter recruitment across diverse healthcare systems and cultural settings would improve generalizability and help determine whether the identified demographic and clinical factors predict changes in quality of life over time.

## 5. Conclusions

In this Romanian cohort, poorer menopause-specific quality of life was independently associated with higher BMI, postmenopausal stage, greater comorbidity burden, and use of medication for menopausal symptoms. Women who sought medical care reported greater symptom burden and poorer menopause-specific quality of life than non-seekers, whereas consultation delay was not significantly associated with either outcome among care seekers. The strong correlation between MRS and MENQOL reflects overlapping patient-reported content and was therefore excluded from the adjusted explanatory model. Clinical assessment should integrate symptom burden, quality of life, general health, and treatment context. Because of the cross-sectional design, these findings do not establish causality or treatment effectiveness.

## Figures and Tables

**Table 1:** Participant characteristics (N = 204).

Variable	Value
Age, years	51 (48–55)
BMI, kg/m^2^	25.4 (22.7–28.4)
Recruitment source: hospital/clinical setting	78 (38.2%)
Recruitment source: community	126 (61.8%)
Urban residence	146 (71.6%)
University education	132 (64.7%)
Married/partnered	159 (77.9%)
Early perimenopause	61 (29.9%)
Late perimenopause	46 (22.5%)
Postmenopause	97 (47.5%)
Years since menopause	2 (0–5.25)
Any comorbidity	86 (42.2%)
Menopause medication use	43 (21.1%)
SSRI or anxiolytic use	21 (10.3%)
Sought medical help	120 (58.8%)
Delay to consultation among care seekers, months	6 (2–12)

Continuous variables are presented as median (interquartile range [IQR]); categorical variables are presented as number (percentage). Abbreviations: BMI, body mass index; IQR, interquartile range; SSRI, selective serotonin reuptake inhibitor.

**Table 2:** Menopausal Symptom Burden and Menopause-Specific Quality of Life.

Variable	Median (IQR)
MRS_total	17.0 (10.0–24.0)
MRS_somatic	6.0 (4.0–8.25)
MRS_psychological	5.0 (3.0–9.0)
MRS_urogenital	5.0 (2.0–7.0)
MENQOL_total	3.66 (2.59–4.66)
MENQOL_vasomotor	2.67 (1.00–5.00)
MENQOL_psychosocial	2.86 (1.71–4.71)
MENQOL_physical	3.88 (2.72–4.77)
MENQOL_sexual	4.33 (1.00–6.00)

Abbreviations: IQR, interquartile range; MENQOL, Menopause-Specific Quality of Life Questionnaire; MRS, Menopause Rating Scale.

**Table 3:** Healthcare-seeking behavior according to menopausal symptom burden and menopause-specific quality of life.

Variable	No Help Sought (n = 84)	Help Sought (n = 120)	*p*-Value
Age, years	52.0 (48.75–56.0)	50.0 (47.75–54.0)	0.053
MRS total	14.5 (7.75–22.0)	18.0 (11.75–24.0)	0.041
MRS psychological	4.0 (2.0–9.0)	6.0 (3.0–9.25)	0.048
MRS somatic	6.0 (3.0–9.0)	7.0 (4.0–8.0)	0.345
MRS urogenital	4.0 (1.75–6.25)	5.0 (2.0–7.0)	0.067
MENQOL total	3.26 (2.30–4.35)	3.95 (2.91–4.79)	0.009
MENQOL vasomotor	2.00 (1.00–4.33)	3.33 (1.33–5.33)	0.014
MENQOL psychosocial	2.57 (1.43–4.00)	3.29 (2.00–4.86)	0.008
MENQOL physical	3.50 (2.36–4.58)	4.19 (3.02–4.89)	0.024
MENQOL sexual	4.00 (1.00–5.67)	4.50 (1.92–6.08)	0.299

Abbreviations: MRS, Menopause Rating Scale; MENQOL, Menopause-Specific Quality of Life Questionnaire; n, number of participants; IQR, interquartile range. Values are presented as median (IQR). We compared groups using the Mann–Whitney U test.

**Table 4:** Correlations between menopausal symptom burden, menopause-specific quality of life, healthcare delay, and treatment satisfaction.

Variables	Spearman’s ρ	95% CI	*p*-Value
Symptom burden and quality of life			
MRS total ↔ MENQOL total	0.858	0.817 to 0.890	<0.001
MRS somatic ↔ MENQOL total	0.730	0.659 to 0.789	<0.001
MRS psychological ↔ MENQOL total	0.723	0.650 to 0.783	<0.001
MRS urogenital ↔ MENQOL total	0.687	0.607 to 0.754	<0.001
Healthcare utilization			
MRS total ↔ Delay to consultation	0.136	−0.045 to 0.308	0.141
MENQOL total ↔ Delay to consultation	0.078	−0.104 to 0.254	0.401
Treatment satisfaction			
MRS total ↔ Treatment satisfaction	−0.311	−0.430 to −0.181	<0.001
MRS somatic ↔ Treatment satisfaction	−0.402	−0.511 to −0.280	<0.001
MRS psychological ↔ Treatment satisfaction	−0.191	−0.320 to −0.055	0.006
MRS urogenital ↔ Treatment satisfaction	−0.215	−0.342 to −0.080	0.002
MENQOL total ↔ Treatment satisfaction	−0.259	−0.382 to −0.126	<0.001

Abbreviations: CI, confidence interval; MENQOL, Menopause-Specific Quality of Life Questionnaire; MRS, Menopause Rating Scale; ρ, Spearman’s rank correlation coefficient. We calculated consultation-delay correlations only among women who sought medical care and provided an interpretable delay value. We assessed treatment satisfaction using a nonvalidated, study-specific single item with five ordinal response categories ranging from 1, very dissatisfied, to 5, very satisfied. The reliability and construct validity of this item are unknown; therefore, correlations involving treatment satisfaction are exploratory and should not be interpreted as precise estimates of a validated satisfaction construct or as evidence of treatment effectiveness.

**Table 5:** Multivariable linear regression model of demographic and clinical factors associated with MENQOL total score.

Variable	B Coefficient	HC3 95% CI	HC3 *p*-Value	VIF
Age, years	−0.035	−0.092 to 0.022	0.225	1.65
BMI, kg/m^2^	0.064	0.006 to 0.122	0.032	1.09
Late perimenopause vs. early perimenopause	0.348	−0.170 to 0.866	0.187	1.54
Postmenopause vs. early perimenopause	0.747	0.200 to 1.294	0.008	2.12
Comorbidity count	0.209	0.026 to 0.392	0.025	1.02
University education	−0.083	−0.498 to 0.333	0.696	1.08
Urban residence	−0.005	−0.458 to 0.448	0.983	1.13
Married/partnered	0.446	−0.085 to 0.978	0.099	1.06
Hospital/clinical recruitment	−0.262	−0.640 to 0.117	0.174	1.11
Menopause-medication use	0.563	0.125 to 1.001	0.012	1.18
SSRI/anxiolytic use	0.525	−0.114 to 1.164	0.106	1.12

Model statistics: R^2^ = 0.184; adjusted R^2^ = 0.138; F(11, 192) = 3.94; *p* < 0.001. B coefficients are ordinary least-squares estimates; confidence intervals and *p*-values are based on HC3 robust standard errors. Early perimenopause, community recruitment, no university education, non-urban residence, not married or partnered, no menopause-medication use, and no SSRI or anxiolytic use were the respective reference categories. Abbreviations: BMI, body mass index; CI, confidence interval; HC3, heteroscedasticity-consistent type 3; MENQOL, Menopause-Specific Quality of Life Questionnaire; SSRI, selective serotonin reuptake inhibitor; VIF, variance inflation factor.

## Data Availability

The dataset used in the study includes sensitive health information at the participant level, collected under informed consent that did not permit public data sharing.

## Abbreviations

The following abbreviations are used in this manuscript:

BMI	Body Mass Index
CI	Confidence Interval
FIGO	International Federation of Gynecology and Obstetrics
GP	General Practitioner
HC3	Heteroscedasticity-Consistent Type 3
IQR	Interquartile Range
IMS	International Menopause Society
MENQOL	Menopause-Specific Quality of Life Questionnaire
MRS	Menopause Rating Scale
NAMS	North American Menopause Society
PROMs	Patient-Reported Outcome Measures
QoL	Quality of Life
SSRI	Selective Serotonin Reuptake Inhibitor
STROBE	Strengthening the Reporting of Observational Studies in Epidemiology
VIF	Variance Inflation Factor
